# Improved access yet inequitable experience: gay, bisexual and other men who have sex with men’s views of more inclusive criteria for source plasma donation

**DOI:** 10.1186/s12889-023-15424-1

**Published:** 2023-04-25

**Authors:** Elisabeth Vesnaver, Terrie Butler-Foster, Mindy Goldman, Emily Gibson, Amelia Palumbo, Don Lapierre, Nolan E. Hill, Richard MacDonagh, Kyle A. Rubini, William Bridel, Glenndl Miguel, Andrew Rosser, Paul MacPherson, Taylor Randall, William Osbourne-Sorrell, Sheila F. O’Brien, Joanne Otis, Mark Greaves, Taim Bilal Al-Bakri, Marc Germain, Shane Orvis, Andrew T. Clapperton, Marco Reid, Maximilian Labrecque, Dana Devine, Justin Presseau

**Affiliations:** 1 Clinical Epidemiology Program, Ottawa Hospital Research Institute, 501 Smyth Road, K1H 8M2 Ottawa, Canada; 2 School of Epidemiology and Public Health, University of Ottawa, Ottawa, Canada; 3 Medical Affairs and Innovation, Canadian Blood Services, Ottawa, Canada; 4 Department of Pathology and Laboratory Medicine, University of Ottawa, Ottawa, Canada; 5 Centre for Sexuality, Calgary, Canada; 6 Local Advisory Group, Calgary, Canada; 7 Local Advisory Group, London, Canada; 8 Faculty of Kinesiology, University of Calgary, Calgary, Canada; 9 Department of Medicine, The Ottawa Hospital, Ottawa, Canada; 10 Department of Biochemistry, Microbiology and Immunology, University of Ottawa, Ottawa, Canada; 11 Department of Sexology, Université du Québec À Montréal, Montreal, Canada; 12 Medical Affairs, Héma-Québec, Quebec City, Canada; 13 Canadian Blood Services, Vancouver, Canada; 14 Centre for Blood Research, University of British Columbia, Vancouver, Canada; 15 School of Psychology, University of Ottawa, Ottawa, Canada

**Keywords:** Integrated knowledge translation, Qualitative, Blood donation, Participatory research, Acceptability, MSM

## Abstract

**Background:**

Canada has incrementally reduced restrictions to blood and plasma donation that impact men who have sex with men, gay, bisexual, and queer men, and some Two Spirit, transgender and non-binary individuals (MSM/2SGBTQ+). Prior to the launch of a pilot program in 2021 enabling some MSM/2SGBTQ + to donate source plasma, we explored the acceptability of the program among individuals who could become eligible to donate in the program.

**Methods:**

We invited men identifying as MSM/2SGBTQ + to participate in two consecutive semi-structured interviews to explore their views on blood and plasma donation policy, plasma donation, and the proposed Canadian plasma donation program. Interview transcripts were analyzed thematically and acceptability-related themes were mapped onto the Theoretical Framework of Acceptability.

**Results:**

Twenty-seven men identifying as having sex with men participated in 53 interviews. Eighteen themes were mapped onto the seven construct domains of the Theoretical Framework of Acceptability. Underlying all aspects of acceptability was a tension between four primary values influencing participants’ views: altruism, equity, supply sufficiency, and evidence-based policy. The program was viewed as welcome progress on a discriminatory policy, with many excited to participate, yet tension with inequitable aspects of the program undermined support for the program and interest to contribute to it. The high demands of the program are unique for MSM/2SGBTQ + and are only tolerable as part of a program that is an incremental and instrumental step to more equitable donation policies.

**Conclusion:**

Findings highlight past experiences of exclusion in Canada as a unique and critical part of the context of the donation experience among MSM/2SGBTQ+. Despite the program’s goals of greater inclusivity of MSM/2SGBTQ + individuals, the anticipated experience of the program included continued stigmatization and inequities. Future research should seek to understand the experienced views of MSM/2SGBTQ + donors to ensure that as policies change, policies are implemented equitably.

**Supplementary Information:**

The online version contains supplementary material available at 10.1186/s12889-023-15424-1.

## Background

In response to a tragedy involving transmission of HIV and hepatitis C through blood transfusion and plasma products in the 1980s, Canada and many other countries followed the lead of the US Food and Drug Administration by introducing restrictions on the donation of blood and blood components by groups identified at the time to be at high-risk of contracting HIV (See [1–3] for overview). Men who have sex with men were one of these groups and were banned from donating if they previously had sex with a man even once since 1977 (the date believed to be the first appearance of AIDS in North America). In Canada there has been much advocacy to change these policies through discourse and research and the restrictions have lessened over the last decade. In 2013, the lifetime ban was reduced to a five-year deferral whereby men were eligible to donate if it had been at least five years since last sexual contact with another man. This time-based deferral was further reduced to one year in 2016 and then to three months in 2019. Nevertheless, these changes still effectively barred men who were sexually active with men from donation.

These criteria are often termed the ‘donor criteria for men who have sex with men’ or ‘MSM donor criteria’. Men who have sex with men (or MSM) is a common category used in epidemiological and public health research. However, *who* is meant to be included in the MSM category varies and the appropriateness of this designation terminology is contested [4–6]. In Canada, at the time of writing, the MSM donor criteria applied to individuals who were assigned male at birth who were sexually active with individuals assigned male at birth unless the individuals in question had undergone lower genital gender affirming surgery[7]. Thus despite a simplistic name, this policy currently impacts many communities including individuals who identify as men who have sex with men, Two Spirit,^1^ gay, bisexual and queer men, transgender and non-binary persons, and other sexual orientations and gender identities not expressly named (hereafter, MSM/2SGBTQ + or *impacted communities*) [8].

In 2021, Canadian Blood Services, the national blood operator outside the province of Quebec, implemented a pilot source plasma donation program in two donor centres (hereafter, MSM plasma program). In the program, persons screened as men who had sex with a man in the previous three months *could* donate source plasma if they had not had a new partner in the last three months and they and their partner only had sex with each other. The pilot program was the first time that men who were presently sexually active with men could donate a blood component (other than for research purposes) in Canada.

Source plasma donation is a type of blood donation that can be made only in certain donation centres in Canada. Plasma is the liquid component of blood and serves in part to transport red and white cells, and platelets. Source plasma is used to produce treatment therapies, such as immunoglobulin, in a process known as fractionation. In a trajectory towards more inclusive criteria, Canadian Blood Services identified source plasma donation as a next step for MSM donor criteria due to the opportunity for additional layers of safety inherent to the fractionation process that is unique to plasma; source plasma can be frozen and quarantined until a donor returns to make another donation.

Under the MSM plasma program, regulators required that a donation would only be released for fractionation when a second plasma donation (made at least 60 days later) tests clear of transfusion-transmissible infections. The rationale given for this strategy was two-fold: (1) Despite great advancements in testing for blood-transmissible infections, there remains a window period whereby early infections are not detectable. The quarantine protocol precludes any possibility of a window period infection. (2) The quarantine enables the collection of evidence on window period infections in a new donor population, which could be used to justify further changes to the policy. Similar programs have been implemented in France and Israel [9, 10]. See [1] and [11] for a review on how other countries have evolved this policy. Although these were the reasons for the MSM plasma program as the next step for MSM donor criteria, these layers of safety are no longer believed to be necessary [12, 13].

Acceptability is a key component of successful implementation of a complex health intervention, such as changes to blood donation criteria [14]. The goal of the MSM plasma program was to implement more inclusive criteria that could enable some men who are sexually active with men to donate source plasma, thereby increasing the donor pool and ultimately increasing Canada’s health resources. UK Medical Research Council’s guidance on developing complex health interventions defines stakeholder engagement as a core element of the development process that should be revisited throughout development, evaluation, and refinement [15]. There is a growing body of literature on the acceptability of different policy options for the MSM donor criteria in Canada, including the acceptability of a plasma donation option [16, 17]. Previous research among Canadian gay, bisexual and other men who have sex with men found that a source plasma-only donation option raised a number of barriers for men to donate [16, 17]. The proposed plasma program in question may raise further barriers due to additional criteria reducing the number of MSM/2SGBTQ + who would become eligible for the plasma-only donation program. Armstrong and colleagues found that interest in blood donation among Canadian gay, bisexual and other men who have sex with men was related to the donor policy conditions under which they would be donating [18]. Even once the policy changes to be more inclusive, the specifics of the policy under which they are eligible to donate make a difference at the individual donor level. Understanding the acceptability of any proposed changes is necessary to enhance the number of eligible donors who would be willing to donate.

This study aimed to explore the acceptability of the MSM plasma program among men impacted by the MSM donor criteria in the two Canadian cities where the program would be implemented. The study was conducted after the program had been submitted for approval to Health Canada (national regulator) but before it had been approved. The MSM plasma program was approved and launched in September 2021.

## Methods

This study is part of a larger multi-method multi-stakeholder study to develop interventions to support source plasma donation among men eligible for the MSM plasma program [19, 20]. We used qualitative methodology to conduct a barrier and enabler assessment among communities impacted by the change in criteria. The acceptability of the MSM plasma program was identified as an important contextual barrier to participation. We then used Sekhon et al’s Theoretical Framework of Acceptability (TFA) to further understand and organize the themes related to acceptability [21]. The TFA was developed to advance the development of the conceptualization of acceptability and in particular to address a demonstrated need for a shared understanding of the components of acceptability and how they can be measured. The TFA is based on a synthesis of systematic reviews of healthcare interventions and describes acceptability as a multi-component construct. Consistency in organization and language related to acceptability can enable policymakers to more easily compare acceptability of different policy options. In this paper, we report on an analysis of views related to acceptability of the MSM plasma program among men identifying as gay, bisexual or as having sex with men in London and Calgary, Canada.

### Methodological approach

Our research approach was rooted in community engagement and integrated knowledge translation. The research team includes research institute- and university-based researchers, community advisors identifying as impacted by the MSM donor criteria, and decision makers and staff from Canadian Blood Services. Integrated knowledge translation involves collaboration with knowledge users that are in positions of power to create change [22]. Our community-engaged approach involved community advisors throughout the research process and we sought to share decision making to ensure that findings benefit the impacted communities [23].

### Study context: donor screening and proposed MSM plasma program

At the time of data collection, donors screened as men were asked “Have you had sex with a man in the last three months?” Sex was defined to include both oral and anal sex. Donors answering yes were not allowed to donate for three months after last sexual contact with a man. These criteria were the same for all blood products and in all Canadian Blood Services donor centres.

The proposed MSM plasma program, set to take place in two donation centres (London, Ontario and Calgary, Alberta), would ask donors screened as men who responded “yes” to having sex with a man two additional questions: (1) “In the last three months, have you had a new sexual partner?” and (2) “In the last three months, have you and your partner only had sex with each other?” Donors with one exclusive sexual partner for at least three months, and who met other standard eligibility criteria, would be able to donate source plasma. Units would be quarantined until a second donation made at least 60 days later tested negative for transfusion-transmissible infections.

### Sample

We used purposive sampling to recruit adult (18 + years) men identifying as gay, bisexual or as having sex with men in London (Ontario) and Calgary (Alberta). Participants were recruited online and with physical posters through our community advisors’ networks, and local organizations and social groups serving MSM/2SGBTQ + communities. Participants were offered a $CAD40 gift card incentive to participate. Twenty-seven individuals who self-identified as gay, bisexual or other men who had sex with men and as being impacted by the MSM criteria participated in the study. Most participants identified as men (> 81.4%), were 30 years or younger (55.6%), were White (> 81.4%), had obtained at least one university degree (81.5%), and were in a relationship (74.1%). See Table 1 for detailed participant characteristics. Total interview time lasted a median of 105 min (IQR = 94.5 to 117.5) and were recorded and transcribed verbatim. Participants were invited to review their transcripts, make edits, and provide additional feedback on their accounts (one participant provided edits).

**Table 1:** Participants Characteristics (N = 27)

Characteristic	Number (*n*)	Proportion (%)
**Age**		
18–30 yrs	15	55.6%
31-40yrs	7	25.9%
>40 yrs	5	18.5%
**Gender**		
Man	> 22 ^a^	> 81.4%
Trans man or without preference	< 5 ^a^	< 18.5%
**Sexual orientation**		
Bisexual, pansexual, queer or multiple	< 5 ^a^	< 18.5%
Gay	> 22 ^a^	> 81.4%
**Ethnicity** ^**b**^		
Hispanic	< 5 ^a^	< 18.5%
Southeast Asian	< 5 ^a^	< 18.5%
White or European	> 22	> 81.4%
Multiracial	< 5 ^a^	< 18.5%
**Education**		
High school or equivalent	0	0%
Certificate or Diploma from a college or University	5	18.5%
Bachelor’s degree	15	55.6%
Degree or certificate above bachelor’s degree	7	25.9%
**In a relationship**		
Yes	20	74.1%
No	7	25.9%
Did not answer	0	0%
**Is the relationship exclusive?**		
Yes	13	65.0%
Yes, mostly	< 5 ^a^	< 25.0%
No, open	< 5 ^a^	< 25.0%

^a^ Exact *n* has been suppressed for participant confidentiality

^b^ Interviewees self-identified their ethnicity.

### Interview procedure

Interviews were semi-structured and designed to be conducted over two sessions. All but one participant completed both interviews. The first topic guide explored the experience of donation, deferral and/or exclusion, understanding about plasma donation, and views on current MSM donor criteria. The second explored participants’ views on the proposed MSM plasma program and possible barriers and enablers to source plasma donation (see Additional File 1 for interview guides). Participants were provided with information about plasma and plasma donation and the rationale for the MSM plasma program. As part of building and maintaining rapport through a sensitive topic guide, “conversational give-and-take” [24] on the history and rationale for the MSM donor criteria was used to build trust with participants. The interviewer was transparent on their personal position that the MSM donor criteria was due for change but did not express specific opinions on the MSM plasma program. Information about plasma and plasma donation was provided and clarified as needed. Interview guides were reviewed and piloted with community advisors prior to data collection. One member of the research team (EV) conducted all interviews. Interviews were conducted from June 2020-December 2021.

### Analysis

Data were analyzed both inductively using thematic analysis [25] and deductively using the Theoretical Framework of Acceptability (TFA) [21]. First, thematic analysis involved open inductive coding of data that was conducted in a systematic fashion. Codes were then refined by comparing data within and across codes to determine similarities and differences. Codes relating to similar topics were abstracted into initial higher order themes. Finally, themes related to acceptability were deductively mapped onto the seven component constructs of the TFA: (1) affective attitude, (2) burden, (3) ethicality, (4) intervention coherence, (5) opportunity costs, (6) self-efficacy, and (7) perceived effectiveness [21]. One member of the research team (EV) conducted all coding and initial mapping. EV met with senior author (JP) to ensure consensus on the interpretation of the themes and how they related to the framework. Feedback was also sought from the community advisory groups. Finally, using drafting the manuscript as a method of inquiry [26], further refinements were made to the mapping which were critically reviewed by all members of the research team.

## Results

**Fig. 1:**
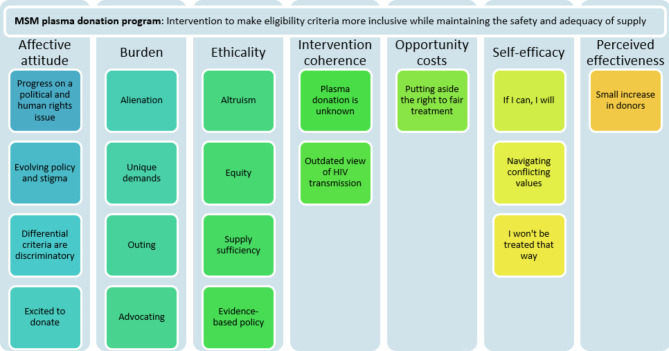
Acceptability of MSM plasma program themes mapped to the domains of the Theoretical Framework of Acceptability (TFA) of healthcare interventions

The thematic analysis generated 19 themes across all domains from the Theoretical Framework of Acceptability (TFA) of health care interventions (Fig. 1).

### TFA construct 1: affective attitude – how did participants feel about the program?

#### MSM plasma program –clear progress on a political and human rights issue

The MSM exclusion criteria for blood donation, or the “blood ban”, as it is commonly referred to among MSM/2SGBTQ + communities, is a political issue. For many, before considering whether they might participate in the program, they considered progress on the rights and freedoms of persons who identify as 2SLGBTQ + and the political promises that had been made by their political leaders to end the ban. Any change on what seemed like a stagnant issue was welcome; “I like the direction it’s [the policy is] heading (P16)”. Progress on the policy itself was important in its own right regardless of participation; “progress is good. If it gets one, three, five more donors, great, there’s a benefit (P23).”

Those not supportive of the program still viewed the step positively if it was an incremental step and not the end state of the MSM donor criteria:

I would generally not be in support of it. However, if it’s a piece with a larger goal, which we’ve talked about it is, I understand that. If it’s one step backwards for two steps forwards, then I would support it in that way, but not on its own, not in isolation, but as part of a larger plan (P25).

Participants integrated the rationale given for the plasma program as an opportunity to collect safety data on donors who identify as MSM/2SGBTQ + into their consideration of the program. The transparency of the scientific rationale for the program, the program’s role in further change to policy and the plan for further changes improved participants’ evaluation of the program:

“if that’s the only way that we can do it within a place that society feels comfortable with, then the ends justify the means […] if that information [the rationale and roadmap] was clear and transparent, that would make me feel better about it (P07).”

For a few participants, a plasma program did not provide meaningful progress to the MSM donor criteria, as one participant said:

…it still makes a distinct policy that gay men are risky…it doesn’t change equity at all. It takes a small portion of men who are in monogamous gay relationships, and let’s them go back. But I don’t know how many people that’s going to affect (P14).

#### Evolving policy and stigma

Participants described that MSM-specific donor criteria makes them feel that their blood is “tainted” or “dirty” and perpetuates societal stigma that sex between men is risky and requires special policy. Regarding the MSM plasma program specifically, some were concerned that a program developed in 2021 that still imposed additional criteria for MSM/2SGBTQ + and required a quarantine for plasma donated in the program further perpetuated this stigma. Furthermore, adding questions about sexual activity and asking these questions only to men with male sexual partners perpetuates the myth that only sex between men is a risk for HIV. One participant explained.

I think it’s quite homophobic and it’s really HIV stigmatising as well. It really kind of comes out there and says, heterosexual people are not going to get HIV, and heterosexual people, you don’t have to ask if they have multiple partners […] Like are these questions founded in good science? Or is this just to make people feel better about having a blood transfusion from a monogamous gay man, like with a white picket fence, living in the suburbs and carrying on this very traditional view of marriage (P07).

Some participants worried that the MSM plasma program would divide MSM/2SGBTQ + communities into those who can and cannot donate based on monogamy. Monogamy as an eligibility criterion was regarded as heteronormative by some participants. Several noted that people in closed relationships with more than one person who are all HIV negative have a similar risk profile yet are excluded from the program. One participant reflected that the separation of MSM/2SGBTQ + along lines of monogamy will perpetuate “the ongoing feeling of judgement and stigmatization of non-traditional relationships and how the queer community kind of pushes those boundaries and still is not accepted and still is looked down upon (P27).” The proposed program risks dividing up their communities into those who are acceptable for donation and those who are not, where acceptability is judged based on their conforming to a value that is viewed as heteronormative by many.

However, this view was not shared by all. Some participants expressed hope that changes to the time-based MSM policy would bring greater awareness to the diversity within the MSM/2SGBTQ + communities. The addition of sexual activity questions in the MSM plasma program represents a move “towards understanding the community a bit better and that there are different practices within relationships (P03)” and acknowledges the existence of relationships between men who are at very low risk of sexually transmitted infections and therefore no increased risk to the blood and plasma supply.

#### Differential criteria are discriminatory

Participants widely held the view that any policy using differential criteria based on gender and sexual orientation is discriminatory. Many participants questioned how such policies were acceptable under Canada’s anti-discrimination laws. The policies were often described as institutionalized discrimination, “one remaining thing that’s actually coded in policy (P24).” As an organization, Canadian Blood Services was discussed as a health care environment due to the nature of the donation process and the presence of health care professionals such as nurses. A health care environment with a policy based on gender and sexual orientation seemed out of step with Canadian legislation to protect the rights and freedoms of people identifying as 2SLGBTQ+.

#### Excited to be allowed to donate

For many, learning about a program that may allow them to donate brought up feelings of excitement. Previous changes in the policy resulted in no practical impact for most MSM/2SGBTQ+. For those in exclusive relationships, the program would open donation for them. Excitement for the approval and launch of the program dominated the discussion because it would allow them personally to donate and donation was very important to them. As one participant said: “It’s a positive step in that, you know, ‘Holy crap, I’m able to actually donate for the first time in my life! (P24).’” Others described very positive experiences of blood donation prior to becoming sexually active with men and were eager to continue this activity, “it would feel good to be able to donate again (P21),” and expressed interest in learning about the process of plasma donation. Some participants described cultures of blood donation in their families and among their circles of friends that they may now be able to participate in. The limitation of plasma was not a barrier: “I’ve of course talked to them after our first interview, and already they were saying, ‘This is so great. As soon as we can, let’s go book a time and go do plasma together (P24).’” They were very keen to donate, even if the opportunity was limited to plasma.

### TFA construct 2: burden – what amount of effort is needed to donate in the MSM plasma program?

For the most part, the MSM plasma program including the additional questions asked of men answering ‘yes’ to having had sex with a man, and the requirement for return donations, were not viewed as burdensome in and of themselves. Many were motivated to donate and keen for the opportunity the MSM plasma program would offer: “I just would be happy that they are willing to ask those questions instead of just immediately dismissing me (P09).” As a result, the differential treatment of the MSM plasma program was not viewed to be burdensome.

The MSM plasma program was only viewed as burdensome due to the injustice that such conditions *were not applied to other donors*. Participants talked about feelings of alienation, unique demands, the stress associated with outing, and the burden of continually advocating for themselves.

#### Alienation

Before the MSM plasma program was explained in detail, participants anticipated positive feelings with donation such as satisfaction and the ‘warm glow’ associated with good deeds. The differential criteria and process involved in the MSM plasma program led participants to anticipate additional feelings of alienation. The program is an indication that they are viewed differently than other donors. As one participant put it: “Here we go. I’m the gay person. So, I have to be put aside and to do that, it plays with your worth. It will never feel good” (P12). Some participants referenced a mistrust of the medical community that is prevalent among MSM/2SGBTQ + communities:

“There would be kind of a feeling of mistrust. When you’re part of this community that already has issues just in your regular life, trying to make sure that you have access to healthcare providers who understand what you do and what you’re going through, that’s not a great feeling.” (P27)

More generally, the program provided a reminder of other experiences of being discriminated because they identify as MSM/2SGBTQ + and reinforcing a sense of alienation in the larger society that they live in. “There’s a lot of stress from being asked a set of questions which are unique to you as a gay man. There’s still some ostracizing happening (P14).”

#### Unique demands

The MSM plasma program involved a number of requirements of MSM/2SGBTQ + donors that were not required of other donors (see Table 2). As one participant asserted: “I’m not interested in jumping through 17 hoops. If you’re going to make this happen [open donation to MSM/2SGBTQ+], then make it happen (P12).”

For most, the unique demands were viewed as burdensome only because they were not required of donors who did not identify as MSM/2SGBTQ+.

I have to go through a longer process because of my sexuality... say yes, I identify as gay or man who has sex with other men, and then all of a sudden, it’s like, ‘Okay, well now we have to have a further discussion - what exactly are you doing? What are your practices? Are you in a relationship? What does that relationship look like?’ (P27)

Some participants noted annoyance at “having” to go to the plasma donor centre rather than a more convenient mobile clinic in their neighbourhood. Others related that maintaining eligibility to make return donations in order to have their past donations utilized placed a burden not only on them personally, but on their partner due to the exclusivity requirement.

**Table 2:** Unique demands of the MSM plasma program not asked of current donors

• Disclosure of sexual orientation • Disclosure of personal details about recent sexual activity • Disclosure of details of one’s personal relationship (exclusivity) • Limited to 1 local location for donation	• Limited to source plasma donation (a longer process than whole blood donation) • Having to make subsequent donations for donations to be utilized • Having to remain eligible in order for past donations to be utilized

#### Outing and hypervigilance

The MSM plasma program invites MSM/2SGBTQ + to donate but requires them to be outed during the screening process. Some participants described a hypervigilance that may be experienced.

When the question comes up, ‘have you had sex with another man in the last three months?’, there’s that kind of time stop or time slowdown in between them asking and wanting to answer, where you start picturing all of their potential reactions. […] I’m on edge, so I kind of interpreted it as a reaction, where there’s like a tsk or a mouth movement. You’re like, wait, what was that? There’s not really a space to ask or reconcile that at all. I can’t ask them, ‘Is that a problem?’ because they’re in a professional position where they’d say, ‘no, no, not at all, of course not’, even if it did kind of feel that way. ‘*Internal trauma*’ for want of a better word (P25).

For some, this hypervigilance is related to the individual to whom the disclosure was made. For others, the hypervigilance extends to the whole donation experience. Participants described feeling that their sexual orientation had made them feel unwelcome in the donor centre. Some anticipated that they would be looking out for any indication that while they may now be allowed to donate as part of the program, they weren’t truly welcome: “I’d be quite sensitive to the interactions with anyone in that space that was sort of proposed that I’m not supposed to be there (P03).” The required outing by the MSM plasma program and the subsequent vigilance for signs of prejudice is an emotional vulnerability and stress not borne by other plasma donors.

#### Advocating

Some participants felt responsible to continually advocate for better policies. This included donating in the program if they could, given the rationale that the data collected in the program was necessary to support better policies. Participants also planned on sharing their views of the inequity of the policy with donor centre staff during a donation. Not only did they feel they had to take every opportunity to advocate for their community, but they also considered the experience of the recipients of their activism.

I feel bad for the nurse, it’s not their policy, they’re just executing what the policy is, and I know my initial inclination is that I’d like to provide some comments back during each opportunity that I would have (P24).

### TFA construct 3: ethicality – How does the MSM plasma program align with participants’ value systems?

**Fig. 2:**
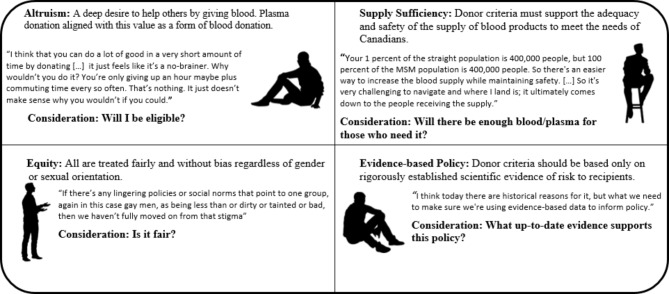
Values participants discussed in relation to their views on the MSM plasma program

The characteristics of participants’ value systems that were most frequently discussed in relation to their views on the MSM plasma program were altruism, equity, sufficient supply, and evidence-based policy (Fig. 2), with altruism and equity being most prevalent. These values were not mutually exclusive among participants, and for some, the MSM plasma program created a conflict of identity as participants needed to preference one value over another in their judgement of the program. For example:

I’d like to say the priority is to increase the supply above all else, but obviously, coming from my point of view and what I’ve experienced […] the most important thing is to remove another thing that’s part of the legacy of discrimination for gay men (P08).

This process of how participants weighed these values in their decisions to participate in the MSM plasma program is discussed under the *TFA construct 6 - Self-Efficacy*.

### TFA construct 4: intervention coherence – do participants understand the intervention and how it works?

Two elements of the MSM plasma program were not clear to participants: plasma donation itself was not well known among participants, and participants disputed that these criteria would adequately safeguard the blood/plasma supply from HIV, particularly related to heterosexual activity.

#### Plasma donation is unknown

Most participants had heard little about plasma donation before participating in the study. They didn’t know what it was used for, yet with a small amount of education most made the logical leap that if it was a type of donation that could be made, then it must be needed and useful. Participants who were strongly motivated by altruism trusted that plasma donations were important and drew from their understanding of and motivation for blood donation to consider the MSM plasma program. “We don’t hear about it [plasma] enough to decide. So, I don’t really have a huge opinion on it. But what I did hear after I saw the post [study recruitment] was very intriguing. And I’m on board(P12).” Others expressed that they would need more information about who and how it helps.

#### Outdated view of HIV transmission

Participants’ perceptions of the MSM donor criteria were that they were mainly meant to protect the supply from HIV. Many accepted that the ban was initiated at a time when HIV was poorly understood, and that HIV was more prevalent among MSM/2SGBTQ + communities. Participants believed that screening for HIV could only be effective when questions about sexual activity were being asked of all donors. “Somebody who is, to use the word *promiscuous*, is also at high risk of HIV, depending on what their activities are. Why men are singled out in that respect is, to me, it’s kind of bizarre (P04).”

Participants either knew that all donations were tested, or they deduced that some back-end technology enabled heterosexual donors to donate without screening their sexual activity. Participants felt that these processes should also be sufficient for donations by MSM/2SGBTQ+.

Given an explanation of the need for donor screening due to the window period for blood-transmissible infections, participants often responded similar to “it’s not like straight people are excluded from having HIV (P14),” since the risk of window period infections would apply to all donors. “I would almost want to reverse argue that to the general public and say, ‘well, aren’t you more concerned with the fact that they’re not screening everybody else?’ (P13)”.

The risk from heterosexual acts is not acknowledged by the current or proposed criteria, yet participants were certain that heterosexual acts carried *some* risk, particularly for those with higher numbers of casual partners. The lack of screening of heterosexual acts contradicted their understanding of HIV transmission and led participants to question the evidence base of the policies.

### TFA construct 5: opportunity costs – The extent to which benefits, profits or values must be given up to donate in the MSM plasma program

#### Putting aside the right to fair treatment

The primary opportunity cost discussed by participants was the requirement to put aside or act against their values of equity. Many felt that participating (and donating) meant implicit agreement with the policy decisions. To participate, they would have to put aside their feelings that the policy, screening, and quarantine were unfair and ultimately put aside their personal right to be treated fairly. One participant described that he would “overlook the back-of-my-mind sentiment that this is unfair (P24).” Discussion on how this opportunity cost contributed to participation decisions is provided under the *TFA construct 6 - Self-Efficacy*.

### TFA construct 6: self-efficacy – How confident are participants that they can participate in the MSM plasma program?

#### If I can, I will

Participants who were primarily driven by their values of altruism were eager to donate whatever they could, whenever they could. “I would be the first to line up to donate for plasma if I was able to (P24).” Many responded to the question of whether they would participate in the MSM plasma program if they were eligible with a resounding and unqualified “yes”. Some participants with strong values of equity were also keen to donate to finally be included where they were not before: “I would probably donate just almost for the sake of, I can now, therefore I will. Like when you get a new right, the idea that you should exercise it (P06).” Given a concrete program that would soon become available, participants who believed they may be eligible were already planning how they would fit regular plasma donation into their lives. Participants who were willing either did not find the program burdensome or they were willing to endure the unique demands:

…[the additional questions are] not saying that you can’t donate, it’s just saying, we need to know a little bit more about you before you can donate. If I was able to do it [donate plasma], then it doesn’t really matter what kind of process I’d have to go through to be able to do that(P15).

#### Navigating conflicting values

Participants discussed their intention to donate as a result of weighing of their values. There was hesitation and pausing as they worked through their feelings. For example.

It would be good to know that you’re helping someone with the plasma even though the policies are still there. So, I’m not sure. I would like to think that I would, but I wouldn’t know for sure. It still feels discriminatory (P22).

It does feel a little bit isolating still, like you’re able to donate, but …almost feels like suspicious of your plasma. Like, “oh, we need another one to really make sure that it is safe”. It feels like a little untrustworthy, which I do understand because it could be like a high stakes thing because it’s given to someone, but yeah…(P09)

For some, learning about the rationale for the MSM plasma program and that their participation may actually contribute to advancing the policy helped to lessen the feeling of inequity and thereby reduce the conflict of values. One participant summarized this integration when answering if they would consider participating: “Knowing what I know through this [interview], absolutely. I feel other people would as well if the communication is managed (P25).”

#### I won’t be treated that way

For some participants, the costs of participating would be too great. One participant had been keen to participate in the MSM plasma program until he learned that any donated plasma would have to be quarantined. He said.

Now you don’t have any of my support. So that’s my answer. […] If you’re only going to use my first sample after...like I fit the criteria and then you are still [..] doing something? No thank you. Have a nice day. I value myself way too much to be treated that way (P12).

For others, participating would mean implicit agreement with the policies, which felt intolerable: “You need to be doing things equitably and properly in order for me to feel comfortable to participate. And until that happens participating it almost feels like you’re sleeping with the devil (P23).”

### TFA construct 7: perceived effectiveness – Would the program bring in more donors who identify as MSM/2SGBTQ+?

#### Small increase in donors

Between limited eligibility among MSM/2SGBTQ + and unwillingness of some who would become eligible to participate, the program’s effectiveness would be reduced in that it would not enable great numbers of new donors identifying as MSM/2SGBTQ+. “I don’t know that gay men, or MSM who are in exclusive relationships who are interested in donating blood, is that large of a population (P11).” It was hard for participants to anticipate how many of those who were interested in the topic were actually interested in donating or because “it was a health organization that seemed like it was discriminating (P20).” Since it is such an important political issue to 2SLGBTQ + communities, some participants questioned if excitement about the change would actually translate to donors in chairs. Others were confident that “the subset who would be eligible would be very ready to donate (P09).”

## Discussion

In this study, we interviewed men who would be impacted by a change in the MSM donor criteria and a new pilot plasma donation program they could become eligible for to explore the program’s acceptability. These findings offer significant insight into the views of the targeted community about an equity-focused policy change and intervention. The novel use of the Theoretical Framework of Acceptability enabled a nuanced description of not only participants’ views, but also the burden of differential treatment, the cost of putting aside a right to equitable treatment, the perceived incoherence of how the program screens HIV, the willingness of participants to donate within the MSM plasma program and the values that underpin their views.

Our study found four primary values that provided the foundation upon which participants considered the MSM plasma program. With varying importance, participants considered the program in light of altruism, equity, supply sufficiency and evidence-based policy. Altruism is an important motivating factor for plasma donation in this population [16] and among current plasma donors [27]. Considerations about equity and the need for policy that is not based on gender and sexual orientation are prominent in other studies examining alternative MSM donor criteria in this population [16, 17] and perception of fairness is an established predictor of policy support more generally [28]. Others have also highlighted the belief that policy should reflect current scientific knowledge about HIV transmission [18, 29]. For some participants for whom evidence-based policy was an important consideration, differential treatment could be justified by scientific evidence and clear communication by the blood operator of the scientific rationale supporting the policies would enhance acceptability. However, to our knowledge, less attention has been paid to supply sufficiency in previous studies. Participants cared about the health of the supply, and some considered the impact of any criteria change on the overall donor pool and current donors. The conflict experienced in these values affected participants’ self-efficacy to participate in the MSM plasma program. Blood operators and policymakers may consider addressing these values as they communicate alternatives to or evolutions of the MSM donor criteria.

Differential treatment was the most problematic aspect of the MSM plasma program. Although it was voiced primarily in relation to the additional screening questions, this may have been due to order effects during the interview. There are several unique costs and burdens to donors in the program. Donors would be outed, asked more personal information, and have to become regular donors in order to contribute. Many altruistically-motivated participants were willing to put aside their values of equity to exercise their altruistic motives. These findings are consistent with other studies, which found that some individuals who are excluded from donation due to the MSM donor criteria would be willing to donate if eligible even under policies they do not feel are fair [16, 30]. In our study, we highlight the internal process that individuals must go through to come to that decision. Programs that are specific to MSM/2SGBTQ + may allow for increased donation opportunities among these communities but as Caruso and colleagues ask, “at what price?” [16]. Why should anyone have to give up fair treatment for themselves and their communities? The anticipated alienation that would be experienced as a result of differential screening policies is particularly critical for policymakers to consider. Alienation exacerbates the minority stress felt in this population and may contribute to negative physical and mental health outcomes [31, 32]. Minority stress refers to the unique stress that individuals from stigmatized social groups experience as a result of their social position. The Meyer minority stress model describes the relationship between stress processes, such as experience of prejudice, expectations of rejection, concealment, and internalized homophobia, and health outcomes [31]. Participants viewed donor centres as health care environments in this study. The experience of alienation within a perceived health care context would worsen the mistrust of health care providers and institutions that is prevalent among MSM/2SGBTQ + communities and may confirm beliefs about how they can expect to be treated in a health care organization [33].

Our data collection differed from previous studies that examined acceptability of plasma-only programs in terms of the scope of education to participants, both regarding plasma and plasma donation, and the rationale for the program. This framing of the program may have enhanced acceptability as presentation of the intervention influences implementation success [34]. Source plasma is not a common type of donation in Canada and donors are typically recruited from regular blood donors. It is unsurprising that it was not well known among a population that has been banned from donating blood [17]. Studies have found that gay, bisexual, and men who have sex with men rated source plasma as a lesser type of donation [16, 17]. However, this was not identified as a primary contributor to the acceptability of the MSM plasma program in the current study. Furthermore, learning that the MSM plasma program would provide the necessary data to support expanding the criteria to blood donation may have reduced the dissonance between participants’ values of altruism and equity. Some participants became not only willing to bear the burdens of the program but eager to encourage others as this activity would now become part of their advocacy efforts in hopes of better conditions in the future. The provision of both a goal for better policy and a plan to get there may have also lessened concerns about the plasma-only nature of the program or that it might be a tactic to end the debate about the MSM donor criteria [16]. Blood donation was concretely on the horizon and the MSM plasma program was only a stopover along the way. Future research should seek to explore the views about plasma being secondary to blood once policies become consistent between plasma and whole blood donation.

Consistent with views on the last change to the MSM donor criteria in Canada [30], the MSM plasma program was viewed as incongruent with current scientific knowledge on HIV transmission and risk reduction. This incongruence fueled views that the program was discriminatory and homophobic and generally reduced its acceptability. Although monogamy is often touted as an irrefutable example of a practice that should justify inclusion in donation regardless of sexual orientation and gender, including only monogamous couples was viewed as heteronormative and insufficiently inclusive of MSM/2SGBTQ + who would be “safe” donors yet whose practices were outside of dominant norms and thus excluded from the program. All participants questioned the absence of acknowledgement in the screening criteria of risk introduced by heterosexual acts, given their knowledge that HIV is also transmitted by heterosexual acts. In Canada, where the source is known, 28% of new HIV infections are due to heterosexual transmission [35]. Communication of how sexually transmitted infections transmitted through heterosexual acts are kept out of the blood and plasma supply may reduce the perception of inequity of the screening and policies because it acknowledges the existence of HIV among heterosexuals, which may in turn reduce the stigma perpetuated by these policies [36].

Participants considered the potential impact of the MSM plasma program on stigma impacting their communities. The public nature of this criteria and debate surrounding it provides broader reach of its impacts. Any policy changes may influence perceptions of the impacted communities among donors who must answer these questions, and within the larger Canadian society. Hofkirchner and colleagues examined bias against MSM/2SGBTQ + depending on the placement of the question about sex with men in the donor screening questionnaire [37]. They compared the placement of the question at the time of the study which was among stigmatizing behaviours (such as intravenous drug use and sex work) to a more neutral placement (within medical history). Although explicit bias was not found to be related to placement, implicit bias was increased for donors answering the MSM question when placed among more stigmatizing behaviours. The authors believed the effect was transitory, as participants were all previous donors and there was no correlation between implicit bias and number of prior donations (and exposure to the placement among stigmatizing behaviours). Nonetheless, these findings highlight the need for policymakers to carefully consider how donor criteria and their operationalization through screening may cause harm. In the case of the MSM plasma program specifically, adding further sexual activity questions *only* for donors identifying as MSM/2SGBTQ + may perpetuate the false and dangerous dichotomization that heterosexual acts are “safe” in contrast to homosexual acts that are “risky”.

Blood operators are under pressure to evolve the MSM donor criteria, and participants emphasized that change in and of itself was positive. This desire for change, even if imperfect, is consistent with other studies in this population [16, 17]. Change demonstrates willingness on the part of the blood operator and the institutions that support its decisions to re-examine past understandings of HIV transmission among their communities, and as a result may reduce the negative stigma perpetuated by these policies. While every step towards more inclusive policy is cause for celebration, our findings highlight that this change is not exclusively positive. Acceptability may be improved if the implicated institutions acknowledge the costs borne by the impacted communities, continue to work towards better policies, and communicate this work back to communities.

Since the collection of this data, a submission by Canadian Blood Services to Health Canada, the regulator of donor criteria in Canada, was approved, and new sexual activity questions will be asked of all donors. All donors (regardless of sexual orientation or gender identity) will be asked about new or multiple partners in the previous three months. Among those who have engaged in these activities in the last three months, those who have had anal sex will not be allowed to donate regardless of condom use. This policy is similar to the sexual activity criteria implemented in the United Kingdom [38]. Critics in Canada have argued that there remains an unwarranted focus on MSM/2SGBTQ + given a focus on anal sex. Heterosexuals who have not engaged in anal sex but who have engaged in other behaviours, such as casual partners and inconsistent condom use, will be allowed to donate. These behaviours are generally perceived among impacted communities as higher risk behaviours regardless of the gender of one’s partner. Indeed, activists argued that the policy implies that **“**a single instance of anal sex (even with condom use and PrEP [pre-exposure prophylaxis] use) carries a greater risk to the blood supply than hundreds or thousands of instances of vaginal sex in the same period” [39], and may reinforce stigmatizing understandings of sex between men as inherently risky. Further work is needed to understand how blood operators can communicate the policy and the rationale in a way that is non-stigmatizing to the individual and does not reinforce stigmatizing beliefs about MSM/2SGBTQ+.

Similarly, impacted communities may feel that blood operators do not understand or take into account the great advances in technology and practices that have reduced the risk of HIV transmission in their communities. Although receptive anal sex with an HIV positive partner without condom use or use of antiretroviral medication by the partner remains the type of sexual activity with the highest risk of HIV transmission, risk estimates drop dramatically when these protective measures are in place, even below that of unprotected vaginal sex with an HIV positive partner [40]. Very high adherence to PrEP is considered to be the most effective protective measure against HIV[41]. PrEP is medication that is preventative of acquiring HIV. It is becoming increasingly accessible in Canada and consequently at the forefront of the community discourse of healthy sexual practices. A policy that limits donation based on having only one partner may come across as disregarding decades of advances in protective practices among impacted communities. This policy addresses the inequitable burden of differential treatment of the MSM plasma program; however, it does not address the incongruence between community understandings of individual risk of HIV transmission during a sexual encounter, sexual health practices, and the rules defining who can and cannot donate. As a result, the policy may not be perceived or experienced as equitable.

Where HIV continues to disproportionately affect MSM/2SGBTQ + communities, Pierik et al. argue that blood operators face morally and ethically challenging choices to develop policies that trade-off on values such as safety to recipient, right to equal treatment including nonstigmatization, and a sufficient blood supply [42]. In the face of policies that continue to disproportionately impact and stigmatize MSM/2SGBTQ+, blood operators have a moral obligation to mitigate the negative impacts on these communities. Our findings highlight opportunities for increased acknowledgement of the harms and costs of these policies, transparency of policymaking and supporting evidence, allyship, and destigmatization of MSM/2SGBTQ+. As policies evolve and new policies are implemented, it will be important to work closely with impacted communities to minimize the harms and costs related to these polices. Doing so may have the added benefit of reducing conflict between the motivating values of equity and altruism, enabling more newly eligible donors to contribute to Canada’s much needed domestic source plasma supply.

### Strengths and limitations

Two-part interviews enabled sustained engagement with participants and facilitated the development of a level of trust necessary to generate rich data [43]. Participants trusted the interviewer when they integrated new information into their consideration of the program and when they demonstrated strong emotions, such as excitement and anger. Serial interviews enabled participants to return to ideas or experiences shared prior and further develop their thoughts resulting in nuanced understanding of the acceptability constructs. Additionally, sharing the findings with the community advisory members of our research team and incorporating their interpretations enhanced the findings’ credibility.

The Theoretical Framework of Acceptability (TFA), enabled reporting of the acceptability of the MSM pilot plasma program along a comprehensive and diverse range of dimensions of acceptability. Building upon the conceptualizations of acceptability in the literature and using consistent language as much as possible enhances opportunities for researchers and policymakers to evaluate and compare programs aiming to implement more inclusive screening. Furthermore, the abstraction to higher level acceptability components enable comparison with other types of donor programs. Although deductive analysis risks losing nuanced understanding of the data [44], the combination of an inductive thematic first phase of the analysis with second phase mapping to the higher order acceptability components enables no loss of information. In this instance, all themes that were developed related to acceptability fit within the TFA, providing support for the TFA as a comprehensive framework. However, not all components of the TFA were well represented by the data, likely due to the fact that the TFA was not used to guide data collection. In particular, opportunity costs were not explicitly explored in interviews. Engaging in plasma donation through this program may mean reduced involvement in other pro-social behaviours [45].

Our recruitment language focused on eliciting opinions about the proposed MSM plasma program and resulted in a highly motivated sample whose views may not represent all communities impacted by the MSM donor criteria. Yet, it is an important group to understand as they would likely be the first to participate if eligible. Similar to the donor pool in Canada and elsewhere [46], the majority of our participants identified as White or of European descent. Certain racial and ethnic groups have additional barriers to donation, such as exclusions based on place of birth, travel, history of malaria, and historical exclusions whose impacts persist long after they have been removed [47]. Our recruitment materials invited gay, bisexual and other men who have sex with men to participate in the study; some materials specifically targeted transmen who have sex with men. These terms enabled inclusion of the population who would be targeted by the MSM plasma program but did not enable inclusion of all communities who would be impacted by the policy. People who identify outside of the gender binary and transfeminine individuals may also be impacted by the MSM donor criteria and MSM plasma program depending on the gender they would be screened in and their sexual activity. Donors who identify as transgender or do not identify on the gender binary face unique barriers to engaging with blood or plasma screening [7]. A more targeted recruitment approach and engagement with communities who are ethnically underrepresented as blood and plasma donors in Canada and/or are gender diverse is needed to comprehensively explore acceptability among all communities impacted.

Participants’ perspectives were collected in anticipation of the implementation of a policy change. It would be important to also assess the acceptability of the policy and its implementation among impacted communities and other implementation outcomes such as the appropriateness of the implementation, the coverage or reach of the implementation, and the sustainability, particularly given the high burden of the plasma quarantine involved in the program [48].

## Conclusion

This study elicited the views and the anticipated experience of the population targeted by a limited-term, equity-focused policy implementation that will open a form of blood donation for the first time in over three decades to some men who are sexually active with men. Four primary values support their views: altruism, equity, supply sufficiency and evidence-based policy. The conflict between the (in)equity of the program and an individual’s drive to engage in an altruistic action impacted their judgements of the program and their decisions about whether they could participate in it. The unique burdens and opportunity costs asked of newly eligible donors identifying as MSM/2SGBTQ + highlighted the potential for an inequitable experience despite more inclusive eligibility criteria. The incongruence of the criteria with current scientific knowledge on HIV transmission and risk reduction, particularly with respect to HIV transmission via heterosexual acts, must be addressed as it continues to fuel the belief that donation policies are not evidence-based. Furthermore, MSM or anal sex-specific policies have the potential to stigmatize MSM/2SGBTQ + communities. There are opportunities to mitigate negative impacts by acknowledging the harms and costs of these policies, communicating the process of policymaking and the supporting evidence, demonstrating allyship, and actively destigmatizing MSM/2SGBTQ+. Future research should seek to understand the experienced views of MSM/2SGBTQ + donors to ensure that policies are implemented equitably as they evolve.

## Electronic supplementary material

Below is the link to the electronic supplementary material.

Supplementary Material 1

## Data Availability

The dataset generated in this current study is not publicly available due to privacy or ethical restrictions. Please contact the corresponding author for further information.

## Footnotes

1 Two Spirit is a term used among some Indigenous persons in North America to refer to a third gender whereby a person embodies both a masculine and a feminine spirit.

## List of abbreviations

TFA: Theoretical Framework of Acceptability
MSM: Men who have sex with men
MSM/2SGBTQ+: Men who have sex with men, Two Spirit, gay, bisexual and queer men, transgender and non binary persons, and other sexual orientations and gender identities not expressly named
PrEP: pre-exposure prophylaxis
