# Supplementary material for: Improved access yet inequitable experience: gay, bisexual and other men who have sex with men’s views of more inclusive criteria for source plasma donation
Source: BMC Public Health. 2023 Apr 25;23:768. doi: 10.1186/s12889-023-15424-1 (PMC10131411; doi:10.1186/s12889-023-15424-1)
Supplement: Supplementary file 1 — Supplementary Material 1 [file 12889_2023_15424_MOESM1_ESM.docx]

**Additional File 1**

**Interview Guide for Expanding Plasma Donation in Canada Study_ community members identifying as gay, bisexual and as having sex with men**

**Notes:**

- For participants in London, we used the terms “gay, bisexual, and other men who have sex with men” or “guys into guys”. For Calgary interviews we used “men who have sex with men”.
- Some additions were made for Calgary (conducted after London) for local preferences and are noted.
- A policy announcement regarding blood and plasma donation eligibility was made during data collection. We added an explanation of the change (described on page 5).

**INTERVIEW #1**

**This first set of questions are to get to know a little more about you so that we can describe in general the guys we have spoken to:**

1. How old are you?
2. How would you describe the area you live in (i.e., urban, suburban, rural,)?
3. How do you identify in terms of gender?
4. What pronouns do you prefer I use for you in the context of this study?^[[1]](#footnote-2)^
5. What’s the highest level of education you’ve completed?
6. How do you identify in terms of race or ethnicity?
7. How did you learn about this study/interview? (What made you want to take part?)

**Now I’m going to ask you a few questions about sexual orientation and relationships**

1. What is your sexual orientation? (gay, bisexual, pansexual, queer, asexual, heteroflexible, straight, prefer to self-describe as __?)
2. In which social circles are you out about your sexual orientation? (at work? At school? At home? With new acquaintances)?
3. Are you currently in a relationship?
   1. Do you mind if I ask you a few questions about your relationship?
      1. How does your partner or partners identify in terms of gender?
      2. How long have you been together?
      3. Do you and your partner (s) only have sex with each other? (i.e. is it monogamous)?

**Lots of people are familiar with blood donation, but today we would like to talk about plasma donation.**

**Before we talk more about it, I’d like to get a sense of what you already know (if anything) about plasma and plasma donation.**

1. What did you know about plasma before taking part in this study?
2. When you think of plasma donation, what thoughts, feelings, or words come to mind (if any)? *Probe*:
3. How familiar are you with the plasma donation process?

*Probe for details to explore different aspects of donation including*:

Do you have a sense of: where you would go to donate? What the screening involves? How long a plasma donation takes? How often donations can be made? Whether you’re currently eligible to donate plasma (and what makes you think this)?

1. When thinking about plasma or plasma donation, is there anything you would want to know more about?
2. Where would you go to find out more about plasma donation?

**Plasma is a part of your blood and makes up 55% of the liquid in your blood. Plasma is like the “transport trailer” of the blood that helps move all the other cells and proteins in the blood around the body. Plasma is mostly water and contains more than 300 different proteins. These proteins are needed to treat serious illnesses and conditions such as trauma, immune disorders or deficiencies, burns and other wounds. The need for these proteins is going up in Canada and increasing plasma collection is a critical public health issue in Canada.**

**Currently, there are specific sites where plasma can be donated. In London, it can only be donated at the donor centre on Wharncliffe road/In Calgary, it can only be donated at the Donor Centre in Eau Claire market.**

**The process of donation starts out like blood donation, the blood is drawn out, then the plasma is separated out and the blood components are returned to the donor. This technique allows for a larger amount to be donated. The whole process takes about an hour and the discomfort and side effects are similar to donating blood.**

**I’d like to ask you some questions now about any experiences you’ve had with plasma or blood donation.**

1. What sorts of experiences have you had with blood or plasma donation (or attempts to donate) in the past?

If past experiences as a donor: How did you get involved as a donor? What was that experience like for you? What are some of the things you considered when deciding to donate?

*Probe for details about the experience:* What type of donation did you make and how often? Where did you make the donations (i.e. at a donor centre, at a blood drive, at school/work)? Are you still currently a donor—if not, what changed? Was donating something you did alone or as part of a group?

*Probe:* Have you ever been denied being able to donate? If so, what was that like?

If no past experiences: What are some things you’ve considered about donating (if any)? Were donor policies about men who have sex with men something that impacted your decision? Was sexual identity or activity ever a factor in your thinking about donating?

1. Have you or anyone you’re close with ever *received* a blood transfusion or plasma product? Tell me about that.

*Probes:* Who was the receiver? What were the circumstances surrounding the transfusion (i.e., emergency or chronic condition, regular need for transfusions versus episodic)? How has [having received/knowing someone who has received a blood product] had an impact on your views about blood/plasma donation?

**An important part of the discussion about blood and plasma donation for men who have sex with men is the blood ban. We recognize that this may upsetting for some to discuss but I think it’s an important piece for us to understand. Would it be alright if I asked you a few questions about it?**

1. What do you know about the role that Canadian Blood Services has in blood donation policies?
2. What do you know about the blood ban in Canada? What thoughts or feelings come to mind when you think about the blood ban?
3. What have you heard about the history around the tainted blood scandal and blood ban?
4. Thinking back, when did you first become aware that men who have sex with men can’t donate blood or plasma?
5. How did you first become aware of it?
6. What was this like for you?
7. What impacts has being excluded from donating had on you (if any)?
8. What impacts do you think these policies have had on the broader LGBTQ+ communities?
9. Have you ever talked about plasma or blood donation screening with colleagues, friends, family members, loved ones? What sort of things have you discussed?
10. Do you think these policies have impacted people’s opinions about Canadian Blood Service or Health Canada?
11. What impacts do you think these policies have had on the blood supply?
12. If eligibility policies were to change,
    1. How would that impact you?
    2. How do you think it would impact the broader LGBTQ+ communities?
    3. In your opinion, how important is blood donation eligibility is for LGBTQ+ communities?
    4. do you think that the legacy of the blood ban would continue to influence people’s motivations to donate?
    5. What do you think can be done to help build trust between blood services and the LGBTQ+ communities?
    6. What are your thoughts on advocacy movements regarding the blood ban? Prompts: protests, boycotts, social media campaigns.
    7. Do you think that Canadian Blood Services should acknowledge the history and experiences of exclusion? If yes, what might that look like to you?

**Policies have started to shift over the last decade but men who have sex with men are still excluded as plasma donors (describe timeline)**

**
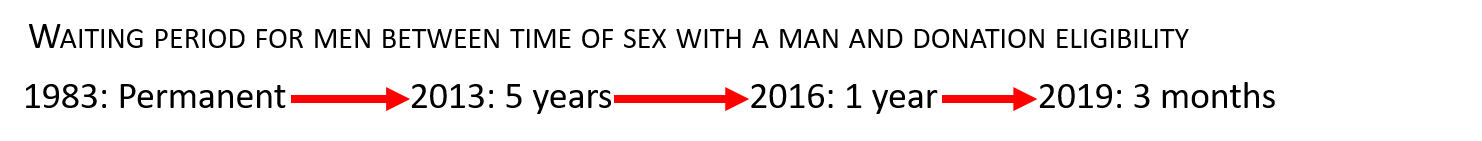
**

1. The current policy is that men are asked during donor screening if they’ve had sex (oral or anal) with a man in the last 3 months. If they answer yes, they cannot donate for 3 months after the date of the encounter.

- 1. What are your thoughts on this screening process and the rules around who can donate?
  2. When you think about being screened as a potential donor under the current policies, how do you feel about it?
  3. What do you think these policies say about the blood of men who are having sex with men? Can you tell me more about this?
  4. What are your thoughts on the current 3 month waiting period from the time of last sexual activity (oral or anal)?

A lot of people have questions about the need for screening when all donated blood and plasma are tested prior to use. The way testing works is that there is a ‘window period’ of new infections when the infection is not yet detectable by the test being used. My understanding from Canadian Blood Services is that with the tests they use, 3 months is the window period they have defined for the sexually transmitted infections they are testing for (hepatitis B & C, Syphilis, & HIV).

- 1. Does thinking about the window period of these infections change your views at all about the current policy and the 3 month- waiting period?
  2. Have your feelings about the exclusion of men who have sex with men changed as the policies have changed over time (i.e. lifetime deferral, 5-year deferral since time of sex with a man, 1-year deferral, to the current 3-month deferral)?

1. I’d like to ask you about the language used in the screening.
2. Donors are identified as men or women based on the donor’s photo ID used for check-in. Donors identified as men are asked in their questionnaire “in the last 3 months, have you had sex with a man?”
   - - - What do you think of how people are identified as men or women?
       - How clear do you find the screening question?
       - Do you have any questions or concerns that come to mind about the wording of the screening question?
       - You have heard me use the categorization of “men who have sex with men” or shortened to MSM to talk about these policies and screening questions. What comes up for you when you think of this categorization?

- Is there any language would you rather see?

1. As of June 2019, having taken PREP in the last 4 months has been added as new exclusion criteria. What are your thoughts on this being included in the screening questions?
2. What are some things that Canadian Blood Services could do to screen donors without contributing to stigma?

**Now we’re going to look forward and discuss changes you’d like to see at Canadian Blood Services or in the donation policies.**

1. When you think about the future of blood & plasma donation in Canada, what would you like to see accomplished?
2. What do you see as the next step towards making plasma donation more inclusive?
   1. How do you think this can best be supported?
   2. What challenges do you think might be faced?
   3. What are your ideas on how to address challenges to inclusion in blood donor policies?
3. What are the most important parts of this conversation to you?
4. As a final question, is there anything that we haven’t touched on today that you think is important to consider on this topic? Prompts: do you have any suggestions for ways to improve the interview process?

Thanks so much for your time and generosity with your thoughts and experiences today. Would you be willing to go ahead with scheduling of the second interview?

**INTERVIEW #2**

**The pilot program that Canadian Blood Services is proposing involves new screening questions that would be asked of men who answer yes to having had sex with a man in the previous 3 months. There is also an additional screening of the plasma that would be donated through this program. Although extremely conservative, the intention was to take a first step towards screening based on risk of sexual activity rather than time since any sexual encounter. In this pilot program, men who are having sex with one partner only would become eligible to donate.**

**CBS has also recently committed to moving to more specific behavioural screening for both blood and plasma donation. However, that will take time to submit and approve. In the meantime, CBS is going ahead with this small plasma donation program in which some gay, bi and queer men will be able to donate.^[[2]](#footnote-3)^**

**Let me back up and describe the current screening process, then I’ll describe what is planned for the pilot. The first layer of screening for everyone is an electronic questionnaire with yes/no questions that is done on a tablet in the centre. It is on this questionnaire where men are asked if they have had sex with a man in the last 3 months. All donors then meet with a registered nurse in a private screening area who reviews the questionnaire. There are a number of the yes/no questions that require additional follow-up questions. Any follow-up questions are asked verbally by the nurse in this private screening area. Donors who are eligible then proceed to the donation area.**

**In the proposed pilot, any man who has answered that he has had sex with a man in the last 3 months on the electronic questionnaire will be asked 2 follow-up questions by the nurse. The questions are:**

1. In the last 3 months, have you had sex with a new partner?
2. In the last 3 months, have you and your partner only had sex with each other?

**Please note that these questions have not yet been approved by Health Canada (although CBS anticipates approval shortly). The choice of these particular questions is based on those used in other countries as well as data generated in earlier parts of this research study and in other Canadian research studies.**

1. How do you feel about the 2 proposed added screening questions for men who have sex with men?
   1. How acceptable do you find the added screening questions (or not)? (prompts: ethically, morally, socially)
   2. How appropriate do you find the added screening questions (or not)?
   3. How clear do you find these questions?
   4. Would you feel comfortable answering these questions during screening? Prompts—is there anything that could make you feel more comfortable answering them
   5. Do you see any challenges with being able to actually answer these questions? [dom bel cap]
   6. Are any of these questions surprising to you?
   7. Were you expecting to see questions that aren’t listed here?
   8. Can you think of any concerns about these added questions?
2. When you imagine being screened using these questions, what feelings or emotions come to mind? [dom. Emotion] (e.g. stress, excitement, nervousness, anger)
3. How do you think the people who are important in your life (e.g. family, friends, colleagues) would feel about this eligibility criteria? [dom. Soc inf]
   1. What about the communities you belong to?
   2. What about public perception and what you hear about in the media?
   3. Whose views are most influential or important to you?
4. Thinking about some of the benefits and drawbacks of the proposed eligibility criteria:
   1. What are some of the benefits or positive aspects of using these additional questions for screening? (Prompts: to you, to those in need of plasma, to LGBTQ+ rights, to the Canadian blood supply)
   2. What are some of the concerns or drawbacks of using these screening questions?
   3. Overall, would this type of screening be positive or negative for you?
5. In your opinion, how ready and willing are men who are having sex with men are to become plasma donors under these criteria?
   1. Overall, how optimistic are you that more men would go to a blood donation centre for screening if these additional questions were being used?
   2. What do you think the introduction of this change should look like?
   3. What could be done to support guys in making a decision to donate or not?
   4. What’s your biggest concern about a possible policy change to include the 2 added screening questions?

**Ok so we talked about the screening for donors, that’s part 1 of the pilot. Part 2 is screening the plasma. This additional screening involves holding a donor’s plasma until they come back for another donation and re-testing of their plasma. The first donation would be held until the donor comes back for their next donation. Upon re-testing (which is done at every visit), the first donation would then get used. This procedure rules out any possibility of an early infection that is not yet detectable (window period infection). This layer has been required by Health Canada.**

1. What would you think of this part of the program?
2. What challenges do you see arising? (for yourself? For other men who are having sex with men?)
3. What ideas do you have for addressing these challenges?
4. If eligible, could you imagine yourself participating in the program? How many times a year could you imagine yourself donating?

Now, while there are many other eligibility criteria, I’d like you to imagine that the current policy restricting men who have had sex with men from donating is no longer in place, and that you are in fact eligible to donate plasma. I know that this is hypothetical, but your answers to this section will identify things that could make it easier or harder to donate other than your feelings about the policy and screening questions.

Plasma can only be donated at the donor centre on Wharncliffe road in London/in the Eau Claire Market Mall in Calgary and the donation process takes about 1 hour from start to finish. The blood is drawn, then the plasma is separated out and the red and white cells are returned to the donor. The current culture of plasma donation is different from that of blood donation. Because plasma donation is something that can be done quite often (unlike blood donation), it’s run more of a program where donors come regularly – and for some it becomes part of their social life. They get to know each other and the staff quite well.

1. On a scale of 1-10 How important is plasma donation to you? How does it compare to blood donation for you?
   1. How would donating plasma fit with your personal values?
   2. What are some things you’d consider when deciding to donate or not?
   3. What would the benefits be for you to donate plasma? Probe in general? To you?
   4. What would the negatives be for you to donate plasma?
   5. Are there any rewarding experiences that would encourage you to donate plasma?
   6. Is there anything else that would motivate you to go to a donor centre to make a plasma donation?
   7. Is there anything you can think of that would dampen your motivation to donate?
2. When you think of making a plasma donation, what sorts of feelings come to mind? (nervous, excited, happy, angry, stressed)
3. Thinking of all the other things you have going on in your life, how much of a priority would donating plasma be for you?
   1. What could make plasma donation easier for you to do?
   2. What would make it harder to do?
   3. Are there any specific resources or materials that would be useful in helping you make a decision about whether or not to donate?
4. How do you think the people who are important in your life (e.g. family, friends, colleagues) would feel about your decision to donate/not donate plasma?
   1. Would you feel supported by those around you to donate?
5. When thinking of going to make a plasma donation, do you foresee any logistical challenges to being able to comfortably make a plasma donation?
   1. Prompts: location of clinic? Travel? Time?

Is there anything about the environment of the donation clinic itself that would make it more or less likely that you would donate plasma (privacy, culture)

1. Is going to make a plasma donation something that you’re likely to think of in your day-to-day life?
   1. What sorts of reminders would be useful?
   2. How would you imagine fitting it into your life?
2. What suggestions do you have for Canadian Blood Services to make plasma donation by men who have sex with men more welcoming?
   1. What signs of openness or inclusion would be meaningful to you (if any)?
   2. When creating materials to support plasma donation, is there any language that you think should be avoided (so words, phrases, questions that could be problematic, hurtful or offensive)?
   3. Is there anything about the CBS webpage or online presence regarding policies for gay, bisexual and other men who have sex with men that you’d like to see changed?
3. [if unwilling to participate in MSM plasma program] Putting your feelings about the proposed plasma donation program aside, If you were eligible, would you intend to donate plasma? How many times a year could you imagine yourself donating?

Out of everything we talked about today about pilot plasma donation program, and about actual donation, what is the most important part of the conversation to you?

**Those are all of my questions for now. Is there anything I didn’t ask you about today that you think should be considered? Is there anything else you’d like to revisit or share?**

**Thank you so much for your generosity of your time, your thoughts and experiences. It really helps us to understand your perspective on these proposed changes to eligibility and how they would be implemented.**

1. Added for Calgary data collection [↑](#footnote-ref-2)
2. Added after announcement June 18, 2021, and included for interviews 23-27. [↑](#footnote-ref-3)
